# Local Phenomena Shape Backyard Soil Metabolite Composition

**DOI:** 10.3390/metabo10030086

**Published:** 2020-02-29

**Authors:** Tra D. Nguyen, Mahbobeh Lesani, Ines Forrest, Yunpeng Lan, Danya A. Dean, Quentin M. R. Gibaut, Yanting Guo, Ekram Hossain, Marcela Olvera, Hannah Panlilio, Adwaita R. Parab, Chaoyi Wu, Jean A. Bernatchez, Robert H. Cichewicz, Laura-Isobel McCall

**Affiliations:** 1 Department of Chemistry and Biochemistry, University of Oklahoma, Norman, OK 73019, USA; 2 Department of Microbiology and Plant Biology, University of Oklahoma, Norman, OK 73019, USA; 3 Skaggs School of Pharmacy and Pharmaceutical Sciences, University of California, San Diego, La Jolla, CA 92093, USA; 4 Center for Discovery and Innovation in Parasitic Diseases, University of California, San Diego, La Jolla, CA 92093, USA; 5 Stephenson Cancer Center, University of Oklahoma, Oklahoma City, OK 73104, USA; 6 Laboratories of Molecular Anthropology and Microbiome Research, University of Oklahoma, Norman, OK 73019, USA

**Keywords:** soil, metabolomics, LC-MS/MS, molecular networking, human activity, natural products

## Abstract

Soil covers most of Earth’s continental surface and is fundamental to life-sustaining processes such as agriculture. Given its rich biodiversity, soil is also a major source for natural product drug discovery from soil microorganisms. However, the study of the soil small molecule profile has been challenging due to the complexity and heterogeneity of this matrix. In this study, we implemented high-resolution liquid chromatography–tandem mass spectrometry and large-scale data analysis tools such as molecular networking to characterize the relative contributions of city, state and regional processes on backyard soil metabolite composition, in 188 soil samples collected from 14 USA States, representing five USA climate regions. We observed that region, state and city of collection all influence the overall soil metabolite profile. However, many metabolites were only detected in unique sites, indicating that uniquely local phenomena also influence the backyard soil environment, with both human-derived and naturally-produced (plant-derived, microbially-derived) metabolites identified. Overall, these findings are helping to define the processes that shape the backyard soil metabolite composition, while also highlighting the need for expanded metabolomic studies of this complex environment.

## 1. Introduction

Soil is a highly complex and diverse mixture of minerals and organic material ubiquitous on the Earth’s surface [1]. Its composition is influenced by large-scale factors such as climate, temperature and humidity, but also local phenomena such as human activity. Soil composition plays an important role in the regulation of many processes, such as plant growth, water systems and microorganism biology [2,3]. The biodiversity and microbial competition in the soil is also a rich source for natural product drug discovery [1,4,5]. Indeed, small molecules (metabolites) are major effectors of biological function, reflecting the active phenotype resulting from an environment’s genetic potential [6]. However, the study of the soil small molecule profile has been limited by the complexity and heterogeneity of this matrix. In recent years, due to its sensitivity and high throughput capabilities, liquid chromatography–tandem mass spectrometry (LC-MS/MS) has become an attractive and powerful analytical tool for targeted and untargeted analysis of metabolite profiles from many sources, including the soil. Targeted LC-MS soil analysis has focused predominantly on the quantification of soil contaminants hazardous to human health, such as pesticides and herbicides, rocket fuel, polycyclic aromatic hydrocarbons and antibiotics [7,8,9,10,11]. Untargeted analyses, in contrast, have focused on environments less impacted by human activity. For example, Ladd et al. developed an untargeted LC-MS/MS method to analyze polar metabolites from an Arctic soil core [12]. Swenson et al. implemented hydrophilic interaction liquid chromatography (HILIC)-MS, across 20 arid biocrust samples from a USA national park, relating metabolite profile following a wetting event to microbial growth [2], while Jenkins et al. studied the metabolite profile of a single soil sample collected from the Oak Ridge Field Research Center by HILIC chromatography [13]. In contrast, Hewavitharana et al. used reversed-phase liquid chromatography to assess the impact of a plant pathogen disinfestation method on an orchard metabolome [14].

Given the importance of soil in agriculture, many of these prior untargeted metabolomic studies have naturally focused on soil samples relevant to this activity. However, the urban ecosystem also has high significance in terms of human health and civil engineering decisions; urban soils may also reflect human activity, although this has yet to be studied by metabolomics [15]. Likewise, given that most untargeted metabolomic studies have focused on a limited number of samples, there is a need for larger-scale metabolomics studies of soils to determine the factors affecting soil composition and assess the metabolic diversity of this environment. To address these gaps, in this study, we leveraged LC-MS/MS and large-scale data analysis tools (principal coordinate analysis, molecular networking [16], MolNetEnhancer [17]). Our goal was to characterize the relative contributions of city, state and regional processes on the backyard soil metabolite composition, and to determine whether this sample type can be used to study human behavior, using samples collected through a crowdsourced citizen science initiative (https://whatsinyourbackyard.org/). Analysis of 188 samples collected from 14 USA States, representing five USA climate regions, led to the detection of 3407 metabolite features, including anthropogenic, plant-derived and microbially-derived metabolites. City, state and regional factors affected the overall metabolite composition, with many metabolite features unique to a given backyard sample. This diversity supports the need for expanded studies of this complex environment using the methodologies implemented here.

## 2. Results

### 2.1. Impact of Collection City, State and Climate Region on the Overall Soil Metabolite Composition

**Figure 1:**
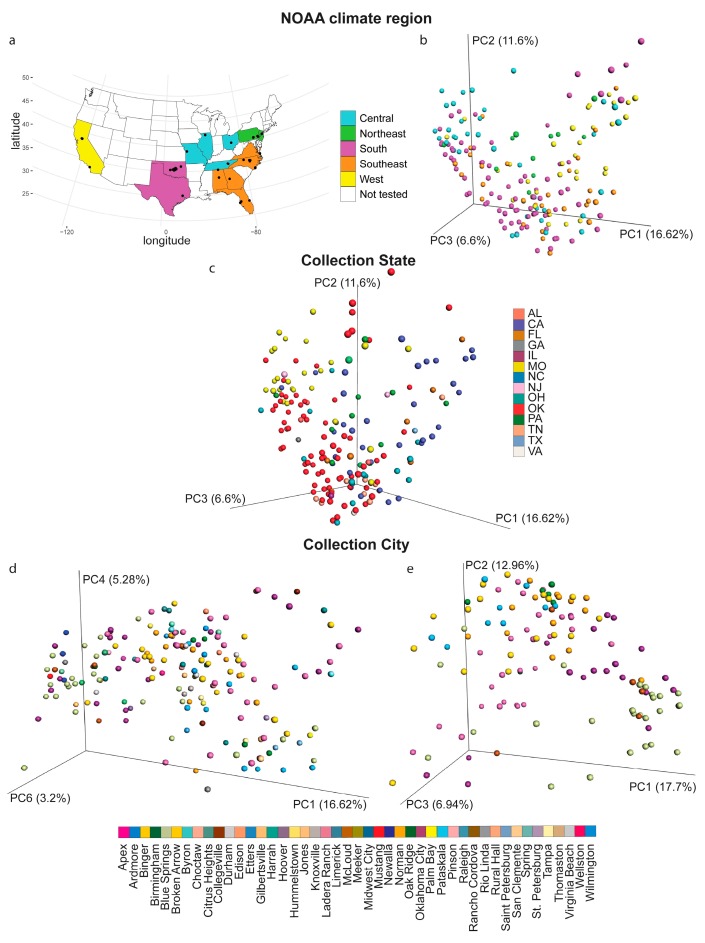
Impact of collection city, state and National Oceanic and Atmospheric Administration (NOAA) region on the overall soil metabolite profile. (**a**) Sampling sites. (**b**) Principal coordinate analysis (PCoA), Bray–Curtis–Faith distance metric, with samples colored by NOAA climate region. (**c**) PCoA analysis, Bray–Curtis–Faith distance metric, with samples colored by state. (**d,e**) PCoA analysis, Bray–Curtis–Faith distance metric, with samples colored by city. (**d**) All samples. (**e**) Analysis restricted to the cities with 5 or more samples (Binger, McLoud, Norman and Oklahoma City, OK; Blue Springs, MO; Ladera Ranch, CA; Oak Ridge, TN; Wilmington, NC).

Metabolites were analyzed from 188 backyard soil samples collected from 45 cities, across 14 states, and representing five of the United States National Oceanic and Atmospheric Administration (NOAA) climate regions (Figure 1a). To determine the relative impact of these geographic factors on the overall soil metabolite composition, principal coordinate analysis (PCoA) was performed. PCoA data showed statistically significant clustering by collection city, state and NOAA climate region, indicating that all these factors influence backyard soil metabolite composition (PERMANOVA analysis, *p* < 0.05 for each metadata category). PERMANOVA analysis of this PCoA data further indicated that NOAA region accounts for 7.38% of the chemical variation in the data, collection state accounts for 15% of the chemical variation and collection city accounts for 33.8% of the chemical variation (Figure 1b, PERMANOVA *p* = 0.001, R^2^ = 0.0738 for NOAA region; Figure 1c, PERMANOVA *p* = 0.001, R^2^ = 0.150 for collection state; Figure 1d, PERMANOVA, *p* = 0.001, R^2^ = 0.338 for collection city). These differences were also apparent when the data were restricted to the cities for which we had the most samples available (Figure 1e, PERMANOVA, *p* = 0.001, R^2^ = 0.203 by city). Overall, collection city, therefore, had the highest effect size, with collection state explaining more of the variation in the data than the NOAA region, but less than collection city. These findings indicate that local phenomena have the strongest impact on backyard soil metabolite profile.

**Figure 2:**
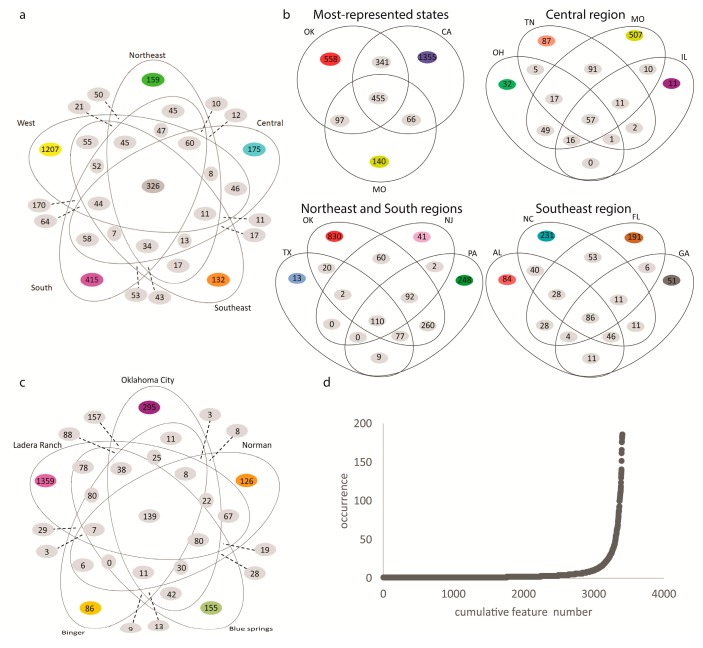
Limited between-sample overlap of detected metabolite features. (**a**) Venn diagram of the detected metabolite features’ distribution across the five sampled regions. (**b**) Venn diagrams of the metabolite features, common or unique, between states. (**c**) Venn diagrams of the metabolite features, common or unique, between cities with ten or more samples: Oklahoma City, Binger and Norman, OK; Ladera Ranch, CA; Blue Springs, MO. (**d**) Rarefaction curve showing that most detected metabolite features only occur in a single sample.

There were 3407 metabolite features retained in our analysis after blank removal. Of those, a core 326 metabolite features were common across all regions (Figure 2a). These include the insect repellent diethyltoluamide (DEET), and plant-derived metabolites (oleanolic acid methyl ester, uvaol and betulinic acid; Table 1, Figure S1). However, when these data were separated by state or city, there was considerable heterogeneity within a region or between cities in a given state (Figure 2b,c). Indeed, 52% of metabolite features were only detected in one soil sample, with 80% in five samples or less (Figure 2d). This indicates that there is still considerable scope for metabolite discovery in soil samples, and highlights the need for large-scale analyses of soil samples.

To determine the local metabolites driving the differences between sampling sites, we focused on Oklahoma, the state for which we had the most samples, and restricted our analysis to cities in Oklahoma with at least ten distinct soil samples analyzed: Norman, Oklahoma City and Binger. Even between these closely-located cities, only 161 overlapping metabolite features were identified (11.7%, Figure 2c). In contrast, 35.5% of metabolite features detected in Binger were not detected in Norman or Oklahoma City, 60% of metabolite features detected in Oklahoma City were not shared with the other two cities and 43.1% of the metabolite features detected in Norman were not detected in the other two cities. Annotatable metabolite features uniquely detected in only one of these three locations compared to the other two cities include human activity-derived metabolites that could reflect differences in behavior between inhabitants of these cities or season of sample collection, as well as plant-derived metabolites that may represent differences in gardening choices between locations. For example, the sunscreen constituent oxybenzone (*m*/*z* 229.086, retention time (RT) 5.92 min, Figure S1) was detected in Oklahoma City but not Norman or Binger (although it was also detected in Ladera Ranch, CA and Blue Springs, MO). Another sunscreen constituent, *m*/*z* 179.070, RT 7.89 min, annotated as 2-propenoic acid, 3-(4-methoxyphenyl), was only found in Oklahoma City (Figure S1). Likewise, the herbicide indaziflam (*m*/*z* 302.177, RT 4.94 min, Figure S1) was only detected in Norman and in no other sampled city. The veterinary anthelminthics oxfendazole (*m*/*z* 316.075, RT 3.43 min) and fenbendazole (*m*/*z* 300.080, RT 4.68 min) were both found in Oklahoma City and not in Norman or Binger, with oxfendazole not detected in any of the other cities we analyzed (Figure S1, Table 1). These differences may reflect pet ownership and differential veterinary or seasonal practices. Several plant-derived metabolites were found at different levels between Binger, Norman and Oklahoma City, including isoliquiritin (*m*/*z* 257.081, RT 4.65 min) and globulol (*m*/*z* 163.148, RT 8.39 min), both detected only in Oklahoma City backyards (Figure S1, Table 1).

To identify metabolites with differential abundance between these three locations, we also built a random forest classifier on metabolites recovered from these locations, classifying by city. Of the top 30 most differential metabolites between Binger, Oklahoma City and Norman (Table S1), only one had an annotation in Global Natural Products Social Molecular Networking (GNPS) that passed our quality criteria (see Methods), the plant metabolite phytol (*m*/*z* 279.304, RT 7.95 min), which was higher in the more rural Binger compared to Norman and Oklahoma City (Kruskal-Wallis *p* = 2.70e−05). Differences in plant-derived metabolites between sampling sites may be due to the types and amounts of plants selected by each household, or to the season of sampling. It is, however, important to note that most metabolites are unique to a given sample (a given backyard) and not shared between multiple locations, even within the same city (Figure 2d).

### 2.2. Specific Chemistries Identified in Backyard Soil Samples

**Figure 3:**
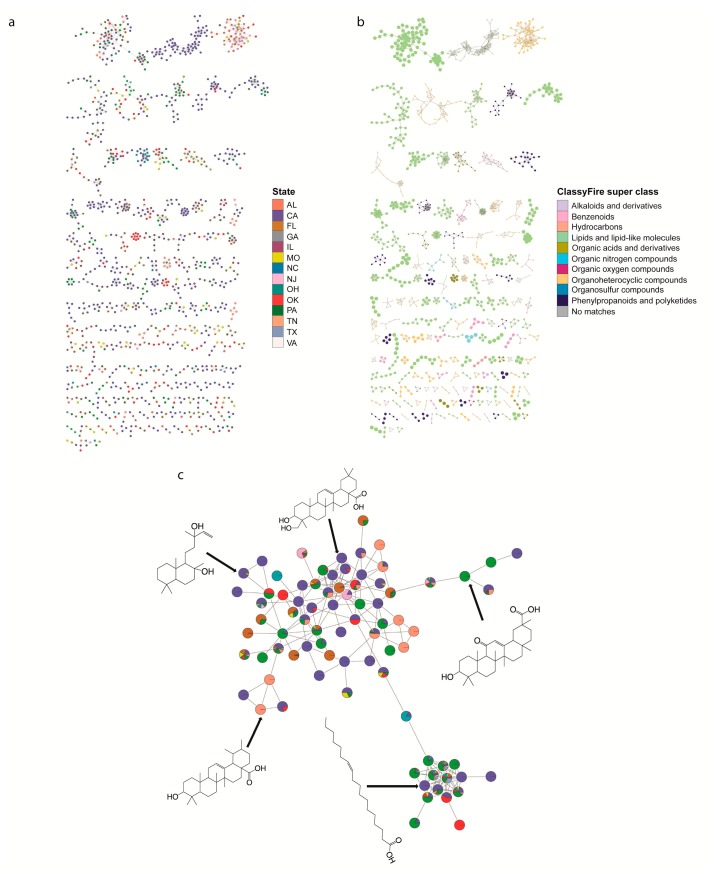
Chemical family analysis of backyard soil metabolites. (**a**) Feature-based molecular networking grouped metabolite features into 171 chemical families. Each node in the network represents one metabolite feature. Nodes connected to each other are structurally-related (cosine MS^2^ similarity score ≥0.7). Nodes are colored by the relative abundance of this metabolite feature between states. (**b**) Molecular network of soil samples colored by 13 selected chemical super classes as indicated in the legend. Node size based on the chemical classification scores for the ClassyFire super class. (**c**) Geographic heterogeneity in diterpenoid and triterpenoid family members. Nodes are colored by the relative abundance of this metabolite feature between states (colors as in panel (**a**)). Five structures of Global Natural Products Social Molecular Networking (GNPS) annotations for this subnetwork are displayed (level 2 annotation confidence [24]; ppm error <1; mass difference <0.001), with arrows pointing to the corresponding node.

To explore the specific metabolites found in backyard soils, we performed feature-based molecular networking [16,18] and chemical ontology analyses of the detected metabolites [17]. Feature-based molecular networking analysis grouped our 3407 detected metabolite features into 171 chemical families (sub-networks) of ≥3 members (Figure 3a), 227 families of two metabolite features (454 network nodes) and 1637 singletons. There was often significant heterogeneity in geographic distribution within a given chemical family. To illustrate this heterogeneity, we focused on terpenes (including triterpenoids and diterpenoids). These are common plant-derived metabolites [19] that were readily annotatable in our dataset. Some triterpenoids were found uniquely in a given state (e.g., *m*/*z* 409.346, RT 7.56 min, annotated as echinocystic acid and found only in CA), while other triterpenoids are found in multiple states and regions (e.g., *m*/*z* 443.389, RT 8.30 min, annotated as uvaol and found in Central, Northeast, West and South regions (OK, PA, CA, TN)) (Figure 3c). Chemical ontology analysis further showed that the soil samples are chemically diverse, with detected features grouped into 13 ClassyFire [20] chemical super classes, 75 classes and 118 subclasses (Figure 3b). The most common chemical superclass was lipids and lipid-like molecules (853 metabolite features), which is consistent with the fact that lipids are commonly found in soils [21,22], and organoheterocyclic compounds (487 metabolite features).

Detected metabolites of interest included plant and microbially-derived secondary metabolites, and small molecules reflecting human activity. Specific plant-derived metabolites included flavonoids (e.g., *m*/*z* 301.107 retention time (RT) 5.20 min, annotated as 5,7-dimethoxy-4′-hydroxyflavanone, or *m*/*z* 285.112, RT 7.14 min, annotated as 5,7-dimethoxyflavanone)*,* triterpenoids (e.g., *m*/*z* 457.368, RT 7.78 min, annotated as betulinic acid or *m*/*z* 409.346, RT 7.56 min, annotated as echinocystic acid) or triterpenoid lactones (e.g., *m*/*z* 455.352, RT 6.26 min, annotated as dehydro(11,12)ursolic acid lactone; Figure 3c, Table 1, Figure S1). Specific microbially-derived metabolites included *m*/*z* 395.367, RT 8.63 min, annotated as fucosterol, or *m*/*z* 462.312, RT 6.80 min, annotated as echinulin, an *Aspergillus* secondary metabolite [23]. Human activity-derived metabolites included sunscreen constituents (e.g., *m*/*z* 179.070, RT 7.89 min, annotated as 2-propenoic acid, 3-(4-methoxyphenyl) and *m*/*z* 229.086, RT 5.92 min, annotated as oxybenzone), insect repellants (e.g., *m*/*z* 192.138, RT 4.61 min, annotated as diethyltoluamide (DEET)), herbicides (e.g., *m*/*z* 282.145, RT 7.18 min, pendimethalin), and medication (e.g., *m*/*z* 278.284, RT 5.77 min, annotated as perhexiline) *(*Table 1, Figure S1).

## 3. Discussion

In this study, we report the metabolomic analysis of 188 soils from across the USA. City, state and NOAA climate region affected the overall metabolite composition, with most metabolite features unique to a given backyard sample (Figure 2d). The impact of climate region on soil metabolites was minor (PERMANOVA R^2^ = 0.0738), indicating that the difference between each region was small in comparison with other phenomena (Figure 1b–e). Source state had a larger impact on soil metabolites (PERMANOVA R^2^ = 0.150), with samples from OK, MO and CA showing partially distinct clustering from other states (Figure 1c). The largest geographic impact on overall soil metabolite profile was observed at the city level (R^2^ = 0.338, Figure 1d,e), indicating that local phenomena explain more of the variation in soil metabolites than broader geography. This was further supported by the considerable heterogeneity in metabolite abundance between locations, even within a given chemical family (Figure 3a,c). Such local factors influencing the soil may include temperature, light radiation, or human factors such as pollution and other human behavior-associated factors. Indeed, several airborne pollutants such as polycyclic aromatic hydrocarbons (PAHs) deposit onto soils [25]. Although our instrumental conditions did not enable us to detect PAHs, we observed many man-made chemicals in our soil samples, including pesticides, insecticides, medication, personal care products and coatings (Table 1).

Roughly 10% of soil metabolite features (326 out of 3407) were, however, observed in at least one sample from each region, indicating a core backyard soil metabolite profile. Indeed, in common with prior soil analyses [21,22,26], we detected high frequencies of lipids (853 metabolite features), as well as amino acids and organic nitrogen compounds. Likewise, similar to studies of root-associated metabolites [27,28,29], we too observed hydroxycinnamic acid derivatives, flavonoids, triterpenoids and other organic acids. Annotatable metabolites detected in our study differed significantly from annotatable metabolites described in prior untargeted analyses of agricultural soils [13,14], likely due to differences in metabolite extraction and instrumental protocols. Overlapping metabolites between the study of agricultural soil by Jenkins et al. [13] and this study include valine and adenosine. A majority of annotatable metabolites in Hewavitharana et al. [14] were lipids, and indeed most annotatable metabolites in our study were lipids and lipid-like molecules (Figure 3b), with pentacyclic triterpenoid derivatives (e.g., ursolic acid) found in both studies. However, these studies of agricultural soils exclusively reported metabolites of natural origin and did not report man-made chemicals. The soil samples we analyzed were collected from backyards and contained many human activity-derived chemicals. These are likely to represent runoff from activities specific to the corresponding household, or to each household’s specific gardening practices. As such, they may represent a fingerprint of household behavior, reminiscent of prior metabolomics studies of the built environment [30,31,32].

A major strength of this study is the large number of samples analyzed from across a broad geographic range, in contrast with many prior soil metabolomics studies (e.g., [12,26]). However, due to the location of this citizen science soil collection project, OK was over-represented compared to other states. Likewise, samples are self-submitted by participants, so that limited metadata is available concerning temperature, weather, plants or human intervention on these soils, even though these are known to affect soil metabolites [2,33]. As with any metabolomics studies, metabolite recovery is affected by experimental procedures. As such, our observations are limited to metabolites soluble in methanol/dichloromethane/ethyl acetate/acetonitrile and ionizable in positive mode. Annotation remains a challenge in metabolomics; indeed, in our dataset, out of 3407 metabolite features, only 55.5% of metabolite features could be assigned to a ClassyFire chemical class, and only one of the most differential metabolites observed between Norman, Binger and Oklahoma City, OK, could be annotated. Nevertheless, our study significantly expands our understanding of soil metabolites, both natural and man-made, and serves as the foundation for further large-scale studies of soil metabolomics.

## 4. Materials and Methods

### 4.1. Sample Selection

Soil samples were obtained from the University of Oklahoma Citizen Science Soil Collection Program (https://whatsinyourbackyard.org/). In total, 188 soil samples were analyzed, representing 45 cities, across 14 states in five of the USA NOAA climate regions (Figure 1a, Table 2).

### 4.2. Metabolite Extraction

Metabolite extraction methods were adapted from previous publications [10,12,34]. Briefly, soil samples were lyophilized overnight. Dried samples were weighed and ca. 50 mg retained for analysis. Dried soils were then pulverized with a 5 mm stainless steel bead (Qiagen), in a TissueLyzer II (Qiagen) set to 25 Hz for 5 min. Pulverized samples were homogenized at 25 Hz for 5 min in methanol/dichloromethane/ethyl acetate/acetonitrile (1:1:1:1 *v*/*v*) spiked with 2 µM sulfachloropyridazine at a 1 mL extraction solvent per 50 mg of soil ratio. Homogenates were shaken in a rotary shaker (Innova 2000, Eppendorf, 150 rpm, 60 min) and then sonicated for 30 min. Finally, the samples were centrifuged (14,800 rpm, 15 min) at room temperature. A 200 μL volume of final supernatant was transferred into 96-well plates to perform LC-MS/MS analysis.

### 4.3. LC-MS/MS

Samples were analyzed in randomized order, with an injection volume of 5 µL. Before each draw, the auto-injector was washed for 2s (10 μL/s) with 10% methanol. Ultra-high performance liquid chromatography was performed on a Thermo Vanquish instrument, using a 1.7 µm Kinetex C18 50 × 2.1 mm column, 100 Å pore size, protected by a SecurityGuard ULTRA C18 Guard Cartridge (Phenomenex), with water + 0.1% formic acid as mobile phase A and acetonitrile + 0.1% formic acid as mobile phase B, at a 0.5 mL/min flow rate. Autosampler temperature was set to 10 °C and column compartment to 40 °C. LC gradient was set according to Table 3.

MS/MS analysis was performed on a Thermo Fisher Q-Exactive Plus hybrid quadrupole orbitrap mass spectrometer. Ions were produced using electrospray ionization and MS spectra acquired in positive mode only. Calibration was done using Thermo Fisher Calmix containing caffeine, MRFA, Ultramark 1621, and n-butylamine in acetonitrile/MeOH/acetic acid solution. Instrument parameters can be found as follows in Table 4.

### 4.4. Data Analysis

Raw MS dataset was converted into mzXML format using MSconvert [35] and imported into MZmine v.2.51 [36]. MS features were identified using parameters described in Table 5.

Data were filtered to remove all metabolite features with intensity within 3-fold of blank samples and normalized to total signal (TIC normalization). Principal coordinate analysis (PCoA) was performed using the Bray–Curtis–Faith dissimilarity metric, using QIIME1 version 1.9.1 [38] and visualized in EMPeror [39]. Global Natural Products Social Molecular Networking platform (GNPS) was used to perform feature-based molecular networking [16,18], with the following parameters: precursor ion mass tolerance of 0.02 Da, fragment ion mass tolerance of 0.02 Da, minimum cosine score of 0.7 and 4 or more matched fragment ions. The maximum shift allowed between two MS/MS spectra was 500 Da, 10 maximum neighbor nodes allowed and the maximum difference between precursor ion mass of searched MS/MS spectrum and library spectra was 100 Da. Molecular network visualization was done in Cytoscape 3.4.0 [40]. Chemical structural information within the molecular network was obtained using the GNPS MolNetEnhancer workflow [17] which incorporated in silico structure annotations from GNPS Library Search, Network Annotation Propagation (NAP) [41] and DEREPLICATOR [42]. DEREPLICATOR was run as part of our feature-based molecular networking job (Advanced external tools: Run Dereplicator enabled). Parameters were therefore kept to defaults: precursor and fragment ion mass tolerance, 0.02 Da; search analogs (VarQuest [43]), enabled; PNP database; max charge, 2; accurate P-values, disabled; minimum number of amino acids, 5. NAP (version 1.2.5) parameters were as follows: N first candidates for consensus score, 10; cosine value to sub-select inside a cluster, 0.8; use fusion result for consensus, enabled; accuracy for exact mass candidate search, 15 ppm; acquisition mode, positive; adduct ion type, [M+H]; structure databases: HMDB, GNPS, SUPNAT, CHEBI; maximum number of candidate structures in the graph, 10; workflow type, MZmine. All annotations are at confidence level 2–3 according to the metabolomics standards initiative [24]. Maps were generated in R using packages muRL, zipcode, ggplot2, mapproj, viridis and RColorBrewer [44]. Random forest analysis was implemented as in our prior work [30], in R using the package randomForest, with 1000 trees and correction for unequal group sizes (see Jupyter Notebook on GitHub at https://github.com/mccall-lab-OU/soils for code details).

### 4.5. Data Availability

LC-MS/MS data is deposited in MassIVE (accession number MSV000084355). Molecular networks can be accessed here: https://gnps.ucsd.edu/ProteoSAFe/status.jsp?task=edfba64fbf5c439ab6057e2aebe2cec5 (full data, feature-based molecular networking), https://proteomics2.ucsd.edu/ProteoSAFe/status.jsp?task=8387016061654f35871327f0c0d7e9cd (NAP), and https://proteomics2.ucsd.edu/ProteoSAFe/status.jsp?task=692ab99133144cb483da6b5959fd66cb (MolNetEnhancer analysis). Jupyter notebooks of data processing and analysis can be accessed at: https://github.com/mccall-lab-OU/soils.

## Figures and Tables

**Table 1:** Representative annotated metabolites.

*m*/*z*	RT (min)	Annotation	Cosine Score	Shared Seaks	Ppm Error	Class or Usage	Region
**Human Activity-Derived Chemicals ^1^**							
121.101	6.97	isophorone	0.94	5	1.70	fertilizer	West
179.070	7.89	2-propenoic acid, 3-(4-methoxyphenyl)-	0.98	6	0.60	sunscreen	South
192.138	4.61	diethyltoluamide (DEET)	0.91	5	2.06	insect repellent, Pesticide	Central, Northeast, Southeast, West, South
229.086	5.92	oxybenzone	0.96	5	0.85	sunscreen	Central, West, South
251.200	6.18	aleuritic acid	0.83	7	0	shellac	West
278.284	5.77	perhexiline	0.88	7	0	vasodilator	Central, South
282.147	7.18	pendimethalin	0.93	9	0	herbicide	Central, South
300.080	4.68	fenbendazole	0.92	5	3	anthelminthic	Southeast, South
302.177	4.94	indaziflam	0.97	7	1	herbicide	South
305.108	6.34	diazinone	0.91	6	0.30	pesticide	Central
316.075	3.43	oxfendazole	0.92	9	0	anthelmintic	South
327.008	5.40	tris(1-chloro-2-propy) phosphate	0.91	4	0.28	adhesives, flame retardants, paint	Southeast, West, South
342.077	5.95	propiconazole	0.96	7	0.89	fungicide	Southeast, West
351.127	7.00	prodiamine	0.85	8	0	herbicide	Central
412.321	6.38	benzethonium	0.91	9	0.22	pesticide, preservative	West
531.408	9.98	didodecyl 3,3’-thiodipropionate oxide	0.96	10	1.72	antioxidant, stabilizer, food preservative	Southeast, South
**Plant-Derived Secondary Metabolites ^1^**							
144.081	7.77	rauwolscine	0.82	5	0	alkaloid	West
163.039	8.17	N-caffeoyl-O-methyltyramine	0.96	6	0	alkaloid	Northeast, Southeast
163.148	8.39	globulol	0.99	7	0.56	sesquiterpenoid	South
201.164	6.77	alpha.-cyperone	0.83	7	0	sesquiterpenoid	West
213.102	4.75	carbanilide	0.98	5	0.43	benzenoid	Central, Northeast, West, South
225.076	8.50	sinapic acid	0.93	9	0.88	hydroxycinnamic acid	Northeast, South
257.081	4.65	isoliquiritin	0.93	7	0	chalcone	South
269.081	4.70	formononetin	0.86	9	0	isoflavonoid	Northeast, West
271.096	5.57	2’,6’-dihydroxy-4’-methoxychalcone	0.99	11	0.68	chalcone	Southeast, West
279.232	6.98	pinoleic acid	0.85	9	0	Fatty acid	Central, West
279.304	7.95	phytol	0.94	8	1	acyclic diterpene alcohol	Central, Northeast, Southeast, West, South
285.112	7.13	5,7-dimethoxyflavanone	0.89	6	0	flavonoid	Northeast
301.107	5.21	5,7-dimethoxy-4′-hydroxyflavanone	0.92	6	0	flavonoid	Southeast
303.232	5.79	isopimaric acid	0.82	13	1	diterpenoid	Central, Northeast
324.170	4.46	(3S,6Z)-3-methyl-6-[[2-(2-methylbut-3-en-2-yl)-1H-indol-3-yl]methylidene]piperazine-2,5-dione	0.82	9	1.22	alkaloid	West
359.149	4.31	matairesinol	0.92	13	0	lignan	West
393.206	6.28	glabrol	0.81	11	0.23	flavonoid	Northeast
407.185	7.01	5,7-dihydroxy-3-(4-hydroxyphenyl)-6,8-bis(3-methylbut-2-enyl)chromen-4-one	0.90	9	0.22	flavonoid	West
409.346	7.56	echinocystic acid	0.86	13	0	triterpenoid	West
409.383	8.56	cycloartenol acetate	0.91	13	0.52	triterpenoid	Northeast, South
411.362	7.78	oleanolic acid methyl ester	0.83	11	0	triterpenoid	Central, Northeast, Southeast, West, South
443.389	8.30	uvaol	0.91	14	0	triterpenoid	Central, Northeast, West, South
455.352	6.26	dehydro (11,12) ursolic acid lactone	0.82	12	0	triterpenoid lactone	Northeast, West
457.368	7.78	betulinic acid	0.81	13	0	pentacyclic triterpenoid	Central, Northeast, Southeast, West, South
**Microbial Metabolites ^1^**							
395.367	8.63	fucosterol	0.85	11	0	sterol	South
462.312	6.80	echinulin	0.86	16	3	diketopiperazine metabolite found in *Aspergillus*	West

^1^ All annotations in this table are at level 2 annotation confidence [24].

**Table 2:** Sample information. Specific collection state and corresponding cities are listed with sample number.

State	Sample Number	City	Sample Number
**Central Region**			
Missouri (MO)	25	Blue Springs	25
Illinois (IL)	2	Bryon	2
Ohio (OH)	2	Pataskala	2
Tennessee (TN)	8	Knoxville Oak Ridge	2 6
Total			37
**Northeast Region**			
Pennsylvania (PA)	10	Collegeville Etters Gilbertsville Hummelstown Limerick	2 2 1 3 2
New Jersey (NJ)	2	Edison	2
Total			12
**South Region**			
Oklahoma (OK)	74	Binger Broken Arrow Choctaw Harrah Jones McLoud Meeker Midwest City Mustang Newalla Norman Oklahoma City Wellston	17 1 2 2 2 5 1 4 2 3 14 20 1
Texas (TX)	2	Spring	2
Total			76
**Southwest Region**			
North Carolina (NC)	15	Apex Durham Raleigh Rural Hall Wilmington	1 3 1 1 9
Alabama (AL)	6	Ardmore Birmingham Hoover Pinson	3 1 1 1
Florida (FL)	7	Palm Bay Saint Petersburg Tampa	2 3 2
Georgia (GA)	2	Thomaston	2
Virginia (VA)	1	Virginia Beach	1
Total			31
**West Region**			
California (CA)	32	Citrus Heights Ladera Ranch Rancho Cordova Rio Linda San Clemente	1 27 1 2 1
Total			32

**Table 3:** LC gradient.

Time	Flow (mL/min)	%B
0.00	0.500	5.0
1.00	0.500	5.0
9.00	0.500	100.0
11.00	0.500	100.0
11.500	0.500	5.0
12.500	0.500	5.0

**Table 4:** Instrument parameters.

Properties of Full MS/dd-MS^2^	
**General**	
Runtime	0 to 12.5 min
Polarity	Positive
Default Charge	1
Inclusion	-
Exclusion	On (see Table S2 for full exclusion list: ions present at 1E5 or higher in extraction blanks were excluded)
Full MS	
Resolution	70,000
AGC target	1 × 10^6^
Scan range	70 to 1050 *m*/*z*
Maximum IT	246 ms
dd-MS^2^	
Resolution	17,500
AGC target	2 × 10^5^
Maximum IT	54 ms
Loop count	5
TopN	5
Isolation window	1.0 *m*/*z*
Fixed mass	-
(N)CE/stepped	NCE: 20, 40, 60
dd Settings	
Minimum AGC	8.00e3
Peptide match	Preferred
Exclude isotopes	on
Dynamic exclusion	10.0 s
ESI Ion Source	
ID	HESI
Sheath gas flow rate	35 L/min
Auxiliary gas flow rate	10 L/min
Sweep gas flow rate	0 L/min
Spray voltage	3.80 kV
S-lens RF level	50 V
Capillary temperature	320 °C
Auxiliary gas temperature	350 °C

**Table 5:** MZmine processing parameters.

Procedure		Parameter
Mass Detection	MS level 1: Noise level	2E5
	MS level 2: Noise level	0.0
	Mass detector	Centroid
ADAP Chromatogram Builder [37]	Min group size # of scans	5
	Group intensity threshold	2E5
	Min highest intensity	5E5
	*m*/*z* tolerance	0.003 *m*/*z* (or 10 ppm)
Chromatogram Deconvolution	Algorithm	Baseline cut-off
	Min peak height	5.0E5
	Peak duration range (min)	0.02–2.2
	Baseline level	2E5
	*m*/*z* center calculation	MEDIAN
	*m*/*z* range for MS^2^ scan pairing (Da)	0.01
	RT range for MS^2^ Scan Pairing (min)	0.1
Isotopic Peaks Grouper	*m*/*z* tolerance	0.001 *m*/*z* (or 10 ppm)
	Retention time tolerance (absolute: min)	0.1
	Monotonic shape	No
	Maximum charge	3
	Representative isotope	Lowest *m*/*z*
Join Aligner	*m*/*z* tolerance	0.001 *m*/*z* (or 10 ppm)
	Weight for *m*/*z*	1
	Retention time tolerance (absolute: min)	0.2
	Weight for RT	0.1
	Require same charge state	Yes
Feature List Row Filter	Retention time (min)	0.25–12.00
	Keep only peaks with MS^2^ scan (GNPS)	Yes

## Supplementary Materials

The following are available online at https://www.mdpi.com/2218-1989/10/3/86/s1, Table S1: Top 30 most differential metabolites between Binger, Norman and Oklahoma City. Table S2: Exclusion list. Figure S1: Metabolite feature annotation support. (a) *m*/*z* 121.101, RT 6.97 min (top, black) match to isophorone library reference (bottom, green). (b) *m*/*z* 179.070, RT 7.89 min (top, black) match to 2-propenoic acid, 3-(4-methoxyphenyl) library reference (bottom, green). (c) *m*/*z* 192.138, RT 4.61 min (top, black) match to diethyltoluamide (DEET) library reference (bottom, green). (d) *m*/*z* 462.312, RT 6.80 min (top, black) match to echinulin library reference (bottom, green). (e) *m*/*z* 229.086, RT 5.92 min (top, black) match to oxybenzone library reference (bottom, green). (f) *m*/*z* 251.200, RT 6.18 min (top, black) match to aleuritic acid library reference (bottom, green). (g) *m*/*z* 278.284, RT 5.77 min (top, black) match to perhexiline library reference (bottom, green). (h) *m*/*z* 282.145, RT 7.18 min (top, black) match to pendimethalin library reference (bottom, green). (i) *m*/*z* 300.080, RT 4.68 min (top, black) match to fenbendazole library reference (bottom, green). (j) *m*/*z* 302.177, RT 4.94 min (top, black) match to indaziflam library reference (bottom, green). (k) *m*/*z* 305.108, RT 6.34 min (top, black) match to diazinone library reference (bottom, green). (l) *m*/*z* 279.304, RT 7.95 min (top, black) match to phytol library reference (bottom, green). (m) *m*/*z* 316.075, RT 3.43 min (top, black) match to oxfendazole library reference (bottom, green). (n) *m*/*z* 327.008, RT 5.40 min (top, black) match to tris(1-chloro-2-propyl)phosphate library reference (bottom, green). (o) *m*/*z* 342.077, RT 5.95 min (top, black) match to propiconazole library reference (bottom, green). (p) *m*/*z* 351.127, RT 7.00 min (top, black) match to prodiamine library reference (bottom, green). (q) *m*/*z* 412.321, RT 6.38 min (top, black) match to benzethonium library reference. (r) *m*/*z* 531.408, RT 9.98 min (top, black) match to didodecyl 3,3′-thiodipropionate oxide library reference (bottom, green). (s) *m*/*z* 144.081, RT 7.77 min (top, black) match to rauwolscine library reference. (t) *m*/*z* 163.039, RT 8.17 min (top, black) match to N-caffeoyl-O-methyltyramine library reference. (u) *m*/*z* 163.148, RT 8.39 min (top, black) match to globulol library reference. (v) *m*/*z* 201.164, RT 6.77 min (top, black) match to alpha.-cyperone library reference. (w) *m*/*z* 213.102, RT 4.75 min (top, black) match to carbanilide library reference. (x) *m*/*z* 225.076, RT 8.50 min (top, black) match to sinapic acid library reference (bottom, green). (y) *m*/*z* 269.081, RT 4.70 min (top, black) match to formononetin library reference (bottom, green). (z) *m*/*z* 271.096, RT 5.57 min (top, black) match to 2′,6′-dihydroxy-4′-methoxychalcone library reference (bottom, green). (aa) *m*/*z* 279.232, RT 6.98 min (top, black) match to pinolenic acid library reference (bottom, green). (ab) *m*/*z* 285.112, RT 7.14 min (top, black) match to 5,7-dimethoxyflavanone library reference (bottom, green). (ac) *m*/*z* 301.107, RT 5.21 min (top, black) match to 5,7-dimethoxy-4′-hydroxyflavanone library reference (bottom, green). (ad) *m*/*z* 303.232, RT 5.79 min (top, black) match to isopimaric acid library reference (bottom, green). (ae) *m*/*z* 324.170, RT 4.46 min (top, black) match to (3S,6Z)-3-methyl-6-[[2-(2-methylbut-3-en-2-yl)-1H-indol-3-yl]methylidene]piperazine-2,5-dione library reference. (af) *m*/*z* 359.149, RT 4.31 min (top, black) match to matairesinol library reference (bottom, green). (ag) *m*/*z* 393.206, RT 6.28 min (top, black) match to glabrol library reference (bottom, green). (ah) *m*/*z* 407.185, RT 7.01 min (top, black) match to 5,7-dihydroxy-3-(4-hydroxyphenyl)-6,8-bis(3-methylbut-2-enyl)chromen-4-one library reference (bottom, green). (ai) *m*/*z* 409.346, RT 7.56 min (top, black) match to echinocystic acid library reference (bottom, green). (aj) *m*/*z* 409.383, RT 8.56 min (top, black) match to cycloartenol acetate library reference (bottom, green). (ak) *m*/*z* 411.362, RT 7.78 min (top, black) match to oleanolic acid methyl ester library reference (bottom, green). (al) *m*/*z* 443.389, RT 8.30 min (top, black) match to uvaol library reference (bottom, green). (am) *m*/*z* 455.352, RT 6.26 min (top, black) match to dehydro (11,12)ursolic acid lactone library reference. (an) *m*/*z* 457.368, RT 7.78 min (top, black) match to betulinic acid library reference (bottom, green). (ao) *m*/*z* 395.367, RT 8.63 min (top, black) match to fucosterol library reference (bottom, green). (ap) *m*/*z* 257.081, RT 4.65 min (top, black) match to isoliquiritin library reference (bottom, green).
