# Supplementary figures and images for: Local Phenomena Shape Backyard Soil Metabolite Composition
Source: Metabolites. 2020 Feb 29;10(3):86. doi: 10.3390/metabo10030086 (PMC7143036; doi:10.3390/metabo10030086)

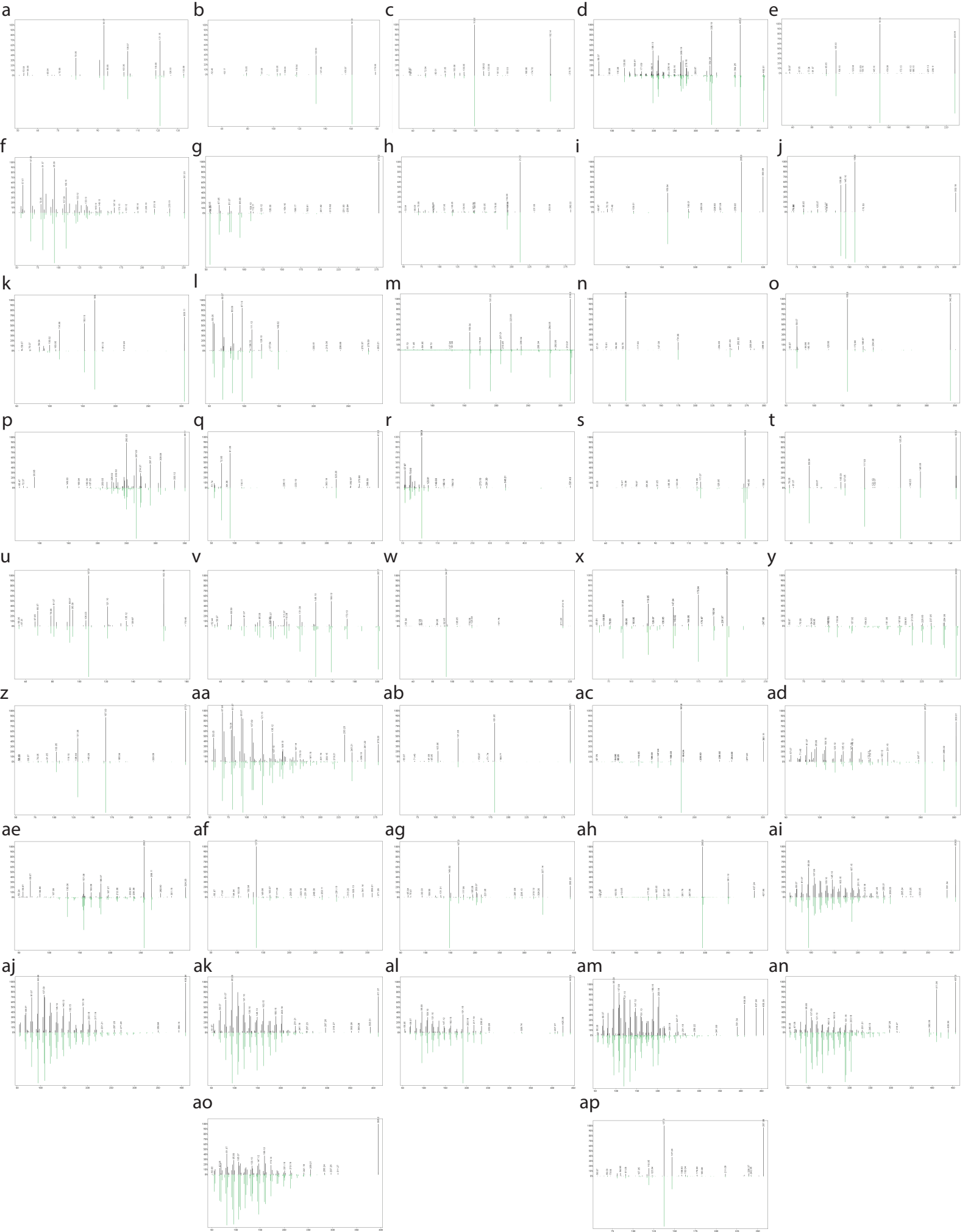

Supplement: Supplementary file 1 [file metabolites-10-00086-s001.zip › Supp info/figS1_modified copy.pdf]
